# Impact of single or repeated short-term heat challenges mimicking summer heat waves on thermoregulatory responses and performances in finishing pigs

**DOI:** 10.1093/tas/txaa192

**Published:** 2020-10-13

**Authors:** David Renaudeau

**Affiliations:** PEGASE UMR1348, Physiologie, Environnement et Génétique pour l’Animal et les Systèmes d’Élevage, INRAE, Institut Agro, St Gilles, France

**Keywords:** heat stress, heat waves, pigs, recovery, thermoregulation

## Abstract

The objectives of this study were to determine the effects of single or repeated short heat stress (HS) challenges that mimicked summer heat waves on performance and thermoregulatory responses in finishing pigs. A total of 45 crossbred castrated males were tested in three consecutive replicates of 15 pigs. Within each replicate, pigs were assigned to one of five treatments. Pigs in treatment group TTT were maintained in thermoneutral conditions (22 °C) for the entire experiment (45 d). Pigs in treatment group HHH were subjected to an HS challenge (32 °C for 5 d) at 113, 127, and 141 d of age (in experimental periods P1, P2, and P3, respectively). Pigs in treatment groups HTT, THT, and TTH were subjected to the HS challenge at 113, 127, or 141 d of age, respectively. Each 5-d challenge was preceded by a 3-d prechallenge period and followed by a 7-d recovery period. Pigs were housed in individual pens and fed ad libitum. HS significantly reduced average daily feed intake (ADFI) and the average daily gain (ADG). Expressed as a percentage of the performance observed during the prechallenge period, ADFI decreased by 12%, 22%, and 26% and ADG decreased by 12%, 43%, and 72% in the HTT, THT, and TTH groups, respectively. Regardless of the experimental group, no compensatory performance was observed during the recovery period, suggesting that HS has a long-lasting effect on animal performance. Pigs subjected to HS had an immediate increase in core body temperature (T_core_), skin temperature, and respiratory rate, all of which gradually decreased during the HS challenge. Based on T_core_ measurements, hypothermia was observed during the recovery period in each of the three experimental periods, especially for pigs in the HHH and the HTT groups but only during the first HS cycle. Repeated exposure to HS for the HHH group resulted in heat acclimation responses characterized by a lower increase in T_core_ and lower decrease in ADFI during P2 and P3 than during P1.

## INTRODUCTION

Economic losses in the pig industry due to heat stress (HS) are high in tropical, as well as temperate, countries (Renaudeau et al., 2012). For U.S. swine industry alone, the annual losses due to HS were estimated by Pollman et al. (2010) at nearly 1 billion. It is clear that HS is a current and emerging issue for world pig production (Nardone et al., 2010). Based on the predicted consequences of climate change, regional warming will increase the frequency, intensity, and duration of summer heat waves in many countries. In addition, genetic selection for rapid lean growth increases metabolic heat production and has subsequent negative effects on heat tolerance (Brown-Brandl et al., 2001; Renaudeau et al., 2011).

The effect of chronic HS in swine has been extensively described in the literature (Ross et al., 2015). When compared to other livestock species, pigs are particularly sensitive to HS because their low ability to sweat decreases their ability to lose heat (Renaudeau et al., 2012). In HS conditions, significantly reducing voluntary feed intake is generally considered a main adaptation mechanism to reduce metabolic heat production. This decrease in feed consumption reduces the average growth rate, increases market weight variability, and alters carcass composition (Ross et al., 2015). The effects of acute heat loads due to summer heat waves have not been as widely researched as chronic HS (Renaudeau et al., 2011). The frequency of these extreme heat events has significantly increased over the past decade and has major consequences on livestock performance, especially in the mid-central United States, Australia, and Europe. In practice, weather forecasts allow pig producers to anticipate problems caused by heat waves. However, developing preemptive strategies to alleviate HS that include heat abatement or feeding strategies requires better a understanding of short- and long-term responses to acute stress exposure, as well as the underlying physiological mechanisms.

The objective of this study was to evaluate the impacts of acute thermal challenges that mimicked repeated bouts of heat during the summer months on pig performance and thermoregulatory responses and test whether these responses differ according to the age of the pig and/or the frequency of HS challenges.

## MATERIALS AND METHODS

The experiment was conducted in accordance with French legislation on animal experimentation and ethics (regional committee number C2EA-07).

### Experimental Design

The study was designed to evaluate the effects of acute HS challenge on the performance and thermoregulatory responses of finishing pigs. The experiment was conducted at the experimental facilities of INRAE in Saint-Gilles (INRAE-UE3P). The study included 45 Pietrain × (Large White × Landrace) crossbred castrated males [67.6 ± 5.0 kg live body weight (BW)] and was conducted in three consecutive replicates of 15 pigs. For each replicate, three blocks of five littermates were selected at 95 d of age and moved to an experimental building with two similar climate-controlled rooms with nine and six individual pens, respectively. The individual metal-slatted pens (0.70 × 2.30 m) were similar and were equipped with a feed dispenser and a nipple drinker designed to avoid spilling feed and water. Pigs remained in the climate-controlled rooms for 60 d, which included a 15-d adaptation period and a subsequent 45-d experimental period. This 45-d period was split into three consecutive periods of 15 d (P1, P2, and P3, respectively). The first room (T room) was kept at 22 °C (thermoneutral conditions for pigs) throughout the entire experiment. The second room (H room) was used to challenge the animals. The challenge, which was repeated in P1, P2, and P3, consisted of a 3-d prechallenge period (22 °C), a 5-d HS challenge (32 °C), and a 7-d recovery period (22 °C). On the first day of the HS challenge, the ambient temperature was gradually increased from 22 to 32 °C at a constant rate of 2 °C/h beginning at 0900 h. On the first day of the recovery period, the ambient temperature was decreased from 32 to 22 °C at a constant rate of 4 °C/h beginning at 0800 h. One pig from each litter was assigned to one of five groups. Animals in groups TTT and HHH were housed in the T and H rooms, respectively, for all 45 d of the experiment (Table 1). Animals in group HTT were housed in the H room in P1 and in the T room in P2 and P3. Animals in group THT were housed in the T room in P1 and P3 and in the H room in P2. Finally, animals in group TTH were housed in the T room in P1 and P2 and in the H room in P3.

**Table 1.** Distribution of the five experimental groups between the two climate-controlled rooms [(T)hermoneutral and (H)eat stress] during the three consecutive periods of the experiment

	Experimental groups				
Period	TTT	HTT	THT	TTH	HHH
P1	T	H	T	T	H
P2	T	T	H	T	H
P3	T	T	T	H	H

Pigs had free access to water and were fed ad libitum with a diet based on cereals and soybean meal that contained 176 g/kg of crude protein and 9.70 MJ/kg net energy. Feed was offered twice per day at 0900 and 1630 h. The photoperiod was fixed at 12 h of artificial light (from 0730 to 1930 h). Room temperature and relative humidity were recorded every 5 min using a data logger (EL-USB-2+, DATAQ instruments, Inc., OH) located in the center of the room at 1 m from the floor. Relative humidity was not controlled.

### Measurements

**Figure 1.**
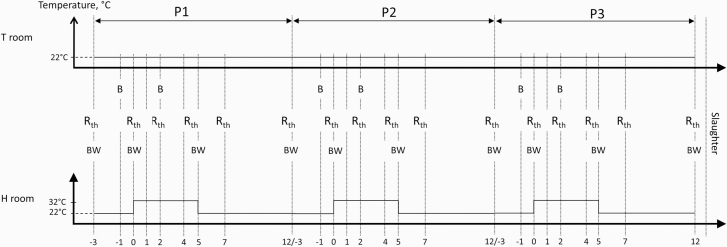
Diagram of measurements performed during the three consecutive periods of the experiment (P1, P2, and P3). Abbreviations: T room, room maintained at thermoneutral conditions (22 °C); H room, room used to challenge the animals (32 °C for 5 d); day 0 = day of temperature change from 22 to 32 °C in the H room (corresponding ages of the pigs were 113, 127, and 141 d for the first, second, and third heat stress challenge, respectively); BW, body weight measurements; Rth, thermoregulatory response measurements (rectal and skin temperatures, respiratory rate); B, blood sampling.

Feed refusals were manually collected each morning at 0800 h and were then weighed and sampled to determine dry matter (DM) content. Feed offered to the animals was sampled weekly to determine DM, and samples were pooled at the end of each replicate for further chemical analysis. Live BW was determined at the beginning and end of P1, P2, and P3 on days −3, 0, and 5 of the HS challenge at a fixed hour (0830 h; Fig. 1). Because the weighing device was located between the two experimental rooms, pigs were transferred from one room to another immediately after each weighing at the end of P1 and P2. During the adaptation period, pigs were familiarized with the weighing system and the transfer between rooms to avoid excessive stress. Ten days before the experimental period began, pigs were surgically implanted with sensors (Anipill, Caen, France) that continually measured internal body temperature (T_core_; once every 2 min; manufacturer accuracy 0.1 °C; resolution = 0.01 °C). Measurements were wirelessly and continuously transmitted to a dedicated recorder. Pigs were anesthetized via intramuscular injection of an anesthetic cocktail of xylazine (2 mg/kg BW) and ketamine (15 mg/kg BW). Following anesthesia, a 2-cm incision was made on the right neck region 5 cm below the ear. The sterile temperature sensor was implanted 4–5 cm into the brachiocephalic muscle. The total duration of the surgical operation did not exceed 10 min. All pigs recovered well and did not develop postsurgical infections. Consequently, none of the pigs received an antibiotic treatment. At the end of the experiment, pigs were slaughtered in INRAE’s experimental slaughterhouse, and the sensor location was checked for signs of infection or inflammation. This surgical procedure was approved by the regional care and use committee (authorization no. 2016022415253973). Rectal and skin (ST) temperatures and respiratory rate (RR) were measured twice per day (0900 and 1600 h) on days −3, 0, 1, 2, 4, 5, and 7 of the HS challenge in P1, P2, and P3 as follows (Fig. 1). First, the RR was visually determined by counting flank movements of resting animals for 1 min. To avoid bias, RR was measured by two experimenters. If their measurements differed by more than 10 breaths per minute, they measured RR again. After completing RR measurements of all pigs, rectal temperature was measured using a digital thermometer (Microlife Corporation, Paris, France). Then, ST was measured on the backs and bellies (flank) using a digital thermometer (HH-21 model, Omega, Stamford, CT) with a K probe.

### Blood Sampling and Chemical Analyses

Feed samples from each period were analyzed for DM, ash, fat content, and crude protein (N × 6.25) according to AOAC (1990) methods. Gross energy content was measured using an adiabatic bomb calorimeter (IKA, Staufen, Germany). Crude fiber content and cell wall components (neutral and acid detergent fiber and acid detergent lignin) were determined according to methods of Van Soest and Wine (1967).

Within each period, blood samples were collected at 0900 h (i.e., 1 h after collecting feed refusals) in restraint animals on days −1 and 2 before meal distribution (Fig. 1). Blood samples (10 mL) were obtained via jugular vein puncture using Vacutainer tubes (Becton Dickinson, San Jose, CA) containing 3.8% sodium heparin as a coagulant. The tubes were then kept on ice for 10 min until centrifugation (10 min at 3,000 rpm), and plasma was immediately subdivided into aliquots and stored at −20 °C. Plasma samples were analyzed for thyroxin (T3) and triiodothyronine (T4). Thyroid hormones were determined using a T3 solid-phase-component system kit and a T4 monoclonal-solid-phase RIA kit (MP Biomedicals, Orangeburg, SC). The coefficients of variation of T3 and T4 intra-assays were less than 1.25% and 7.90%, respectively.

### Calculations and Statistics

**Figure 2.**
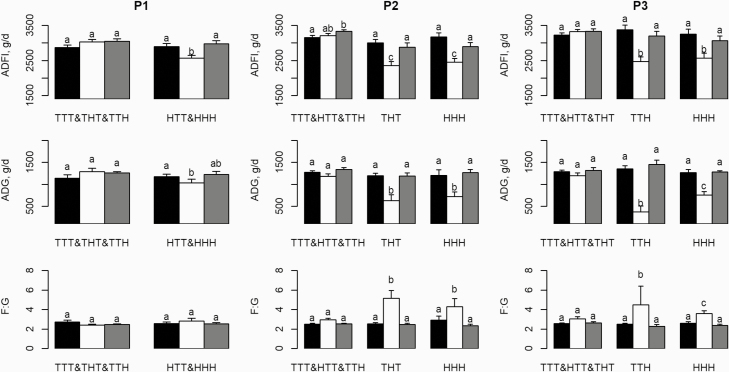
Effects of the temperature treatment and time (−3 ≤ day < 0; black bars, 0 ≤ day <5; white bars, 5 ≤ day < 12; gray bars) on ADFI, ADG, and F:G in the first (P1), second (P2), and third (P3) experimental periods. Error bars indicate the standard error of least square mean (27, 18, 27, 9, 9, 27, 9, and 9 pigs for treatments TTT&THT&TTH, HTT&HHH, TTT&HTT&TTH, THT, HHH, TTT&HTT&THT, TTH, and HHH, respectively). ^a,b,c^: bars with different superscripts differ significantly between the three periods (*P* < 0.05).

Feed intake of each pig was determined from the daily weighing of feed offer and refusal. Then, average daily feed intake (ADFI in g/d or in g/d/kg^0.60^), average daily gain (ADG in g/d), and the feed–conversion ratio (F:G in kg of feed/kg of gain) were calculated for the total duration of the experiment. These data were analyzed in a general linear model using the GLM procedure of SAS (SAS Inst. Inc., Cary, NC), with the fixed effects of experimental group, replicate, and their interaction. Within each period, performance data were split into three time intervals: days −3 to 0, days 0 to 5, and days 5 to 12. In P1, data from the TTT, THT, and TTH groups were pooled into a single group called “T,” while data from the HTT and HHH groups were pooled into a single group called “H.” In P2, data from the TTT, TTH, and HTT groups were pooled into a single group called “TT&HT,” while the THT and HHH groups were renamed “TH” and “HH,” respectively. In P3, data from the TTT, HTT, and THT groups were pooled into a single group called “TTT&HTT&THT.” These data were subjected to a repeated MIXED procedure of SAS, with the fixed effects of experimental group, replicate, time interval, and their interactions. Blood parameters were analyzed with a similar model. ST and RR were first averaged (from the measurements taken at 0900 and 1600 h) and, then, in each period, the effect of the duration of the HS challenge on these thermoregulatory traits was analyzed using a MIXED model, with the fixed effects of experimental group, replicate, time interval, and their interactions. Continual measurements of internal body temperature and ADFI were averaged daily per pig for each of the three periods. According to Renaudeau et al. (2010), pigs have a biphasic thermoregulatory response that consists of initial hyperthermia or hypophagia within the first 24 h of exposure to HS followed by a recovery period characterized by a gradual decrease in body temperature or increase in ADFI. To distinguish changes in ADFI and internal body temperature clearly during the thermal acclimation periods, we used a model adapted from Koops and Grossman (1991), with two “threshold days” (which mark the beginning or intermediate phase of the acclimation response):


$$\begin{array}{ll} Y= & \;\;y_{0}+v_{1}\times d-r_{1}\times \left(v_{1}-v_{2}\right)\\ & \times ln\left[1+exp\left(\left(d-td_{1}\right)/r_{1}\right)\right]r_{2}\times v_{2}\\ & \times ln\left[1+exp\left(\left(d-td_{2}\right)/r_{2}\right)\right]+\varepsilon ij \end{array}$$

where *Y* is the response variable (g/d/kg^0.60^ or °C) from days −1 to 10, *y*_0_ (g/d/kg^0.60^ or °C) is the value of *Y* on day 0, td_1_ and td_2_ (days of exposure) are the threshold days, and *v*_1_ and *v*_2_ (g/d^2^/kg^0.60^ or °C/d) are the linear changes in *Y* before and after td_1_ and after td_2_, respectively (Fig. 2).

In this approach, *r*_1_ and *r*_2_ determine the smoothness of the transition around td_1_ and td_2_, respectively. In the present study, *r*_1_ and *r*_2_ were determined for each variable, with the assumption that it was not influenced by temperature. A nonlinear MIXED model (NLMM) was fitted using the NLMIXED procedure of SAS. It included a random effect related to each animal to reflect the extent to which individual profiles deviated from the overall average profile. Because NLMIXED does not provide adjusted *R*^2^ values, we estimated it as follows (Robbins et al., 2006):


$$\begin{array}{l} Adjusted\;R^{2}=1-\left[SSE/\left(n-p-1\right)\right]/\left[CTSS/\left(n-1\right)\right] \end{array}$$

where SSE is the sum of squared errors (calculated from the estimated residuals), CTSS is the corrected total sum of squares, *n* is the number of observations, and *p* is the number of parameters.

## RESULTS

Pigs remained in good health throughout the experiment, and no medical treatment was administrated through the diet or by injection. In thermoneutral conditions (i.e., T groups and during the prechallenge and the postchallenge periods), actual ambient temperature and relative humidity averaged 22.3 ± 0.4 °C and 54.6 ± 7.5%, respectively. The corresponding values during the HS challenge were 32.9 ± 0.4 °C and 41.7 ± 3.6%, respectively.

### Growth Performance

The experimental treatment significantly influenced growth performance throughout the experiment (Table 2). ADFI and ADG were significantly lower (*P <* 0.05) in the HHH group than in the TTT, HTT, and TTH groups. Intermediate values were observed in the THT group. Mean F:G, carcass dressing rate, and lean content were similar for all treatment groups (2.55 kg/kg, 81.6% and 58.5%, respectively). Compared to the average performance measured before the HS challenge (i.e., from days −3 to 0), ADFI and ADG significantly decreased during the first 5-d HS challenge (−12% for both), while the F:G did not change (mean of 2.58 kg/kg; Fig. 2). During the recovery period (i.e., from day 5 to 12), ADFI and ADG were similar (*P >* 0.05) to those before the HS challenge (2,971 vs. 2,899 g/d, respectively, for ADFI and 1,227 vs. 1,176 g/d, respectively, for ADG). In P2, growth performance significantly decreased during the HS challenge in the THT and HHH groups (Fig. 2). Their mean ADFI and ADG decreased by 22% and 43%, respectively. As observed for P1, no compensatory performance was observed during the recovery period. In THT and HHH groups, F:G significantly increased during the HS challenge (Fig. 2). Compared to the F:G before the HS challenge, the increase was similar in the THT and HHH groups (2.55 vs. 5.15 kg/kg, respectively, in the THT group and 2.90 vs. 4.30 kg/kg, respectively, in the HHH group). In P3, the growth performance of the thermoneutral groups (TTT, HTT, and THT) did not differ (*P >* 0.05; Fig. 2). Compared to the average performance before the HS challenge, the TTH group had a significantly lower ADFI (3,380 vs. 2,473 g/d, respectively; *P <* 0.01), ADG (1,351 vs. 376 g/d, respectively; *P <* 0.01), and a higher F:G (2.50 vs. 4.50 kg/kg, respectively; *P <* 0.05). For pigs with previous HS challenges (the HHH group), compared to before the HS challenge, growth performance also decreased but to a lesser extent than that in the TTH group (3,247 vs. 2,571 g/g, respectively, for ADFI; 1,270 vs. 753 d/g, respectively, for ADG; and 2.60 vs. 3.68 kg/kg, respectively, for F:G).

**Table 2.** Effect of the experimental treatment on growth performance and carcass quality (least square means of nine pigs per group)

	Experimental groups						
Characteristic	TTT	HTT	THT	TTH	HHH	RSD	Statistics^*a*^
BW, kg							
Initial	68.2	69.4	67.6	69.7	68.2	4.3	
Final	122.0	121.4	117.3	121.1	115.5	6.3	
Final^*b*^	123.0^a^	120.9^a^	118.8^ab^	120.1^a^	114.5^b^	3.9	G**
ADFI, g/d	3,218^a^	3,153^a^	2,949^ab^	3,132^a^	2,858^b^	221	G**
ADG, g/d	1,280^a^	1,240^a^	1,184^ab^	1,223^a^	1,090^b^	92	G**
Adj. ADG, g/d^*b*^	1,283^a^	1,238^a^	1,189^ab^	1,220^a^	1,087^b^	93	G**
F:G ratio	2.51	2.54	2.50	2.57	2.62	0.20	
Dressing rate, %	81.7	81.8	81.5	81.5	81.5	1.0	R*
Lean content, %	59.0	57.0	59.7	58.0	59.0	2.2	G^t^

RSD, residual standard deviation.

^*a*^Data were analyzed using a general linear model that included the effect of experimental group (G) and replicate (R) and the interaction G × R as fixed effects.

^*b*^Adjusted for an initial BW of 68.6 kg (mean in the experiment).

^a,b^Least square means in the same row with different superscript letters differ significantly (*P* < 0.05).

^t^ *P <* 0.10, *P < 0.05, **P < 0.01.

**Figure 3.**
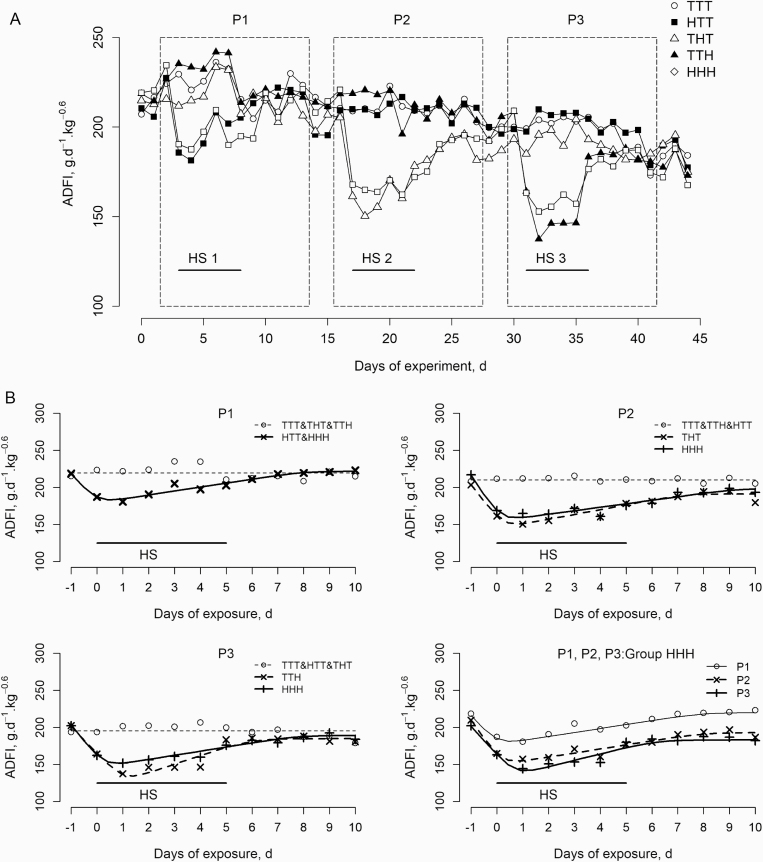
(A) Effects of the experimental treatment (HS challenges) in the first (P1), second (P2), and third (P3) experimental periods on ADFI (g/d/kg^0.60^) profiles throughout the experiment. Each point is the least square mean of nine pigs. (B) ADFI profiles predicted using a nonlinear model for each period for all experimental groups and for all periods for the HHH group. Equation parameters are shown in Table 3. Day 0 is the transition day from 22 to 32 °C.

Dynamics of ADFI (g/d/kg^0.60^) varied among treatments during the experiment (Fig. 3A). Regardless of the period, ADFI was similar (*P >* 0.05) in pigs from groups that were not subjected to the HS challenge (groups TTT, THT, and TTH in P1; TTT, HTT, and TTH in P2; and TTT, HTT, and THT in P3). Conversely, pigs in groups HTT, THT, and TTH exposed to the HS challenge in P1, P2, and P3, respectively, and in group HHH in all three periods, showed a significant decrease (*P <* 0.001) in ADFI as soon as the temperature increased to 32 °C (i.e., on days 3, 17, and 31 of the experiment in P1, P2, and P3, respectively). This initial decrease was followed by a gradual recovery in ADFI over the successive days of HS challenge. ADFI as a function of the duration of exposure to 32 °C was modeled independently for each period or, for the HHH group, for all periods (Fig. 3B). Equation parameters are shown in Table 3. In P1 and the HS groups (i.e., HTT and HHH), ADFI significantly decreased at a rate of 39.1 g/d^2^/kg^0.60^ after the transition from 22 to 32 °C. It then linearly increased at a rate of 5.3 g/d^2^/kg^0.60^ from day 0 to 8 before plateauing at a value (*P >* 0.05) similar to that on day −1. Overall, similar trends were observed for pigs challenged with HS in P2 and P3. In P3, the threshold day when ADFI began to increase (i.e., td_1_) tended to occur sooner in the HHH group than in the TTH group (0.27 vs. 1.03 d, respectively; *P =* 0.10), and the linear increase after td_1_ (i.e., *v*_2_) was significantly higher in the TTH group than in the HHH group (11.56 vs. 5.99 g/d^2^/kg^0.60^, respectively; *P =* 0.04). In the HHH group, repeated HS challenges significantly influenced ADFI patterns, with a later td_1_ (0.71 vs. 0.10 d; *P =* 0.05) and a larger *v*_2_ (8.63 vs. 5.42 g/d^2^/kg^0.60^; *P =* 0.03) in P3 than in P1 or P2 (Table 3). Compared to reference values measured on day −1, ADFI on day 10 was significantly lower in P2 and P3 (−16.7 and −16.6 g/d/kg^0.60^, respectively; *P <* 0.01), while no significant difference was found in P1 (mean of +3.6 g/d^2^/kg^0.60^; *P >* 0.05).

**Table 3.** Parameter values to describe effects of temperature on long-term acclimation responses in growing pigs by treatment group and period of the experiment

			Model parameters												
*Y* ^*a*^	Period	Group	y_0_		v_1_		v_2_		td_1_		td_2_		σ _r_²	σ _e_²	Adj. *R*²
ADFI, g/d/kg^0.60^															
	P1	TTH&HHH	179	(14.6)	−39.1	(0.8)	5.34	(0.84)	0.01	(0.35)	7.97	(1.04)	300	331	0.65
	P2	THT	158^a^	(10.4)	−45.6^a^	(10.9)	6.50^a^	(1.14)	0.27^a^	(0.26)	7.26^a^	(0.86)	249	245	0.67
	P2	HHH	162^a^	(14.5)	−54.5^a^	(14.9)	4.72^a^	(0.80)	0.13^a^	(0.28)	9.30^a^	(1.13)			
	P3	TTH	165^a^	(7.7)	−36.3^a^	(7.1)	11.50^a^	(2.23)	1.03^a^	(0.31)	5.97^a^	(0.24)	295	304	0.68
	P3	HHH	159^a^	(11.4)	−44.3^a^	(12.9)	5.99^b^	(1.62)	0.27^a^	(0.32)	7.76^a^	(1.42)			
T_core_, °C															
	P1	TTH&HHH	39.3	(0.05)	0.48	(0.04)	−0.30	(0.02)	1.46	(0.13)	6.30	(0.20)	0.038	0.036	0.87
	P2	THT	39.2^a^	(0.12)	0.36^a^	(0.07)	−0.39^a^	(0.05)	2.06^a^	(0.30)	5.67^a^	(0.24)	0.057	0.100	0.86
	P2	HHH	39.0^b^	(0.12)	0.50^a^	(0.07)	−0.34^a^	(0.03)	1.43^b^	(0.20)	6.42^a^	(0.38)			
	P3	TTH	39.1^a^	(0.11)	0.41^a^	(0.07)	−0.25^a^	(0.03)	1.34^a^	(0.27)	6.13^a^	(0.35)	0.051	0.087	0.83
	P3	HHH	38.7^b^	(0.12)	0.42^a^	(0.08)	−0.29^a^	(0.04)	1.68^a^	(0.35)	6.33^a^	(0.32)			
ADFI, g/d/kg^0.60^															
	P1	HHH	177^a^	(14.8)	−39.1^a^	(15.4)	5.35^a^	(0.82)	0.00^a^	(0.36)	7.98^a^	(1.08)	324	324	0.70
	P2	HHH	160^a^	(9.2)	−49.8^b^	(10.2)	5.49^a^	(0.78)	0.20^a^	(0.21)	7.99^a^	(0.77)			
	P3	HHH	163^b^	(6.1)	−38.5^a^	(7.2)	8.63^b^	(1.41)	0.71^b^	(0.26)	6.41^a^	(0.59)			
T_core_, °C															
	P1	HHH	39.4^a^	(0.06)	0.48^a^	(0.05)	−0.30^a^	(0.02)	1.45^a^	(0.16)	6.30^a^	(0.23)	0.054	0.102	0.85
	P2	HHH	39.1^b^	(0.07)	0.44^a^	(0.05)	−0.35^a^	(0.03)	1.63^a^	(0.18)	5.98^a^	(0.21)			
	P3	HHH	38.9^c^	(0.07)	0.42^a^	(0.05)	−0.27^b^	(0.02)	1.50^a^	(0.19)	6.25^a^	(0.23)			

^*a*^ADFI (g/d/kg^0.60^) and T_core_ (°C) responses from day −1 to 10 were fit to a nonlinear model: *Y* = *y*_0_ + *v*_1_ d – *r*_1_ (*v*_1_  *+ v*_2_) ln(1 + exp((*d* – td_1_)/*r*_1_) – *r*_2_*v*_2_ ln(1 + exp((*d* – td_2_)/*r*_2_), where *y*_0_ is the value of *Y* at day 0, td_1_ and td_2_ (days of exposure) are threshold days, and *v*_1_ and *v*_2_ are the linear changes in *Y* before and after td_1_, and after td_2_, respectively. σ _*r*_^2^: individual variance for each parameter in the studied population, σ _*e*_^2^: residual variance of the model.

^a, b, c^Within a line, means with different superscripts are significantly influenced by temperature (*P <* 0.05).

### Thermoregulatory Responses

**Figure 4.**
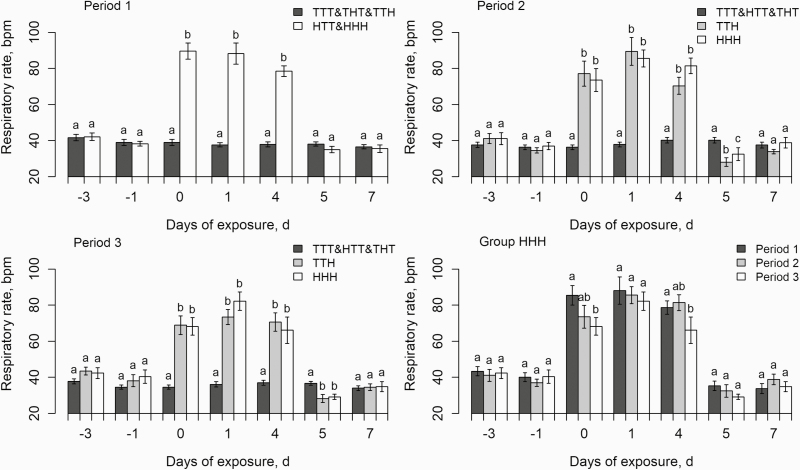
Effects of days of exposure (day 0 = transition day from 22 and 32 °C) and experimental group on respiratory rate. For period 1, each bar is the least square mean of 27 and 18 pigs for nonchallenged (i.e., TTT&THT&TTH) and challenged groups (HTT&HHH), respectively. For period 2, each bar is the least square mean of 27, 9, and 9 pigs for nonchallenged (i.e., TTT&TTH&HTT) and challenged groups (THT and HHH), respectively. For period 3, each bar is the least square mean of 27, 9, and 9 pigs for nonchallenged (i.e., TTT&HTT&THT) and challenged groups (TTH and HHH), respectively. For the HHH group, each bar is the least square means of nine pigs in each experimental period. ^a,b,c^: bars with different superscripts differ significantly between experimental groups for periods 1, 2, and 3 or, for the HHH group, between periods (*P <* 0.05).

**Figure 5.**
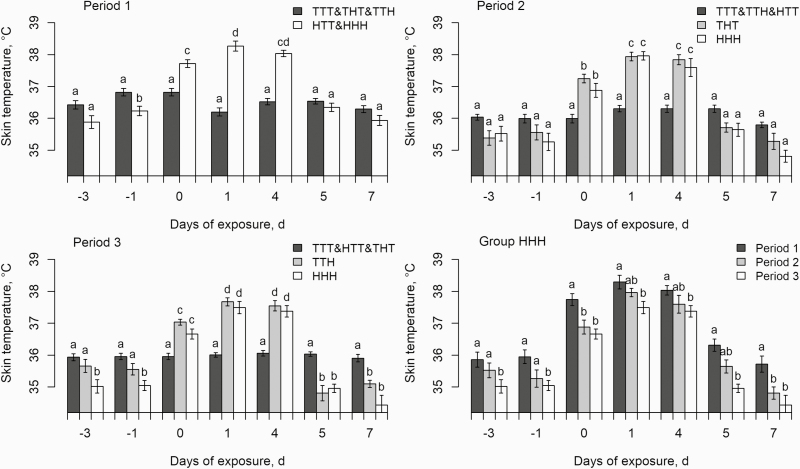
Effects of days of exposure (day 0 = transition day from 22 to 32 °C) and experimental groups on skin temperature. For period 1, each bar is the least square mean of 27 and 18 pigs for nonchallenged (i.e., TTT&THT&TTH) and challenged groups (HTT&HHH), respectively. For period 2, each bar is the least square mean of 27, 9 and 9 pigs for nonchallenged (i.e., TTT&TTH&HTT) and challenged groups (THT and HHH), respectively. For period 3, each bar is the least square mean of 27, 9, and 9 pigs for nonchallenged (i.e., TTT&HTT&THT) and challenged groups (TTH and HHH), respectively. For the HHH group, each bar is the least square means of nine pigs in each experimental period. ^a,b,c^: bars with different superscripts differ significantly between experimental groups in periods 1, 2, and 3 or, for the HHH group, between periods (*P* < 0.05).

**Figure 6.**
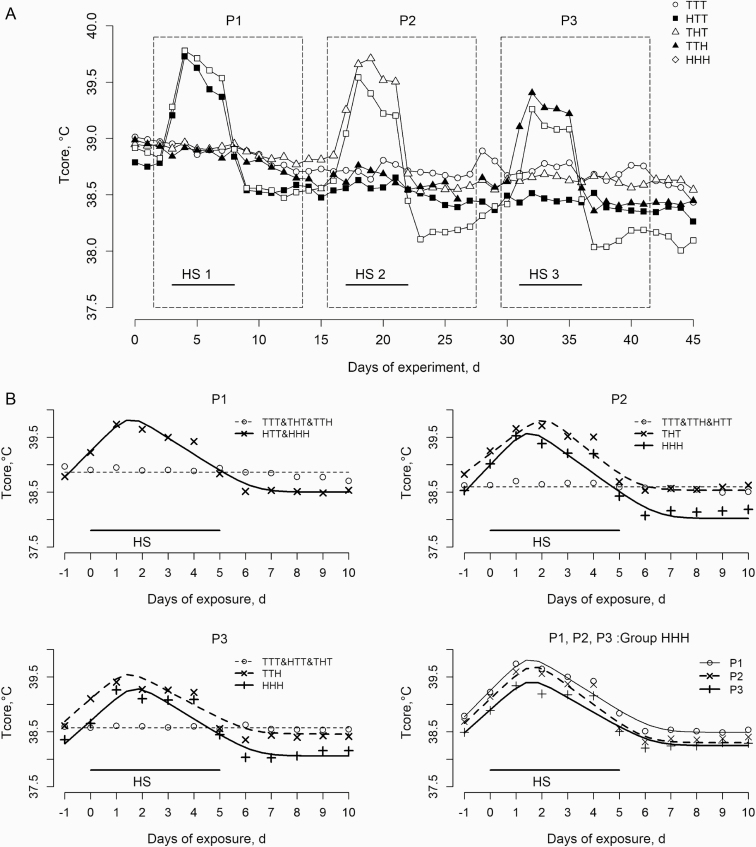
(A) Effects of experimental treatments (HS challenges) in the first (P1), second (P2), and third (P3) experimental periods on internal body temperature (°C) profiles throughout the experiment. Each point is the least square mean of nine pigs. (B) T_core_ profiles predicted using a nonlinear model for each period for all experimental groups and for all periods for the HHH group. Equation parameters are shown in Table 3. Day 0 is the transition day from 22 to 32 °C.

**Figure 7.**
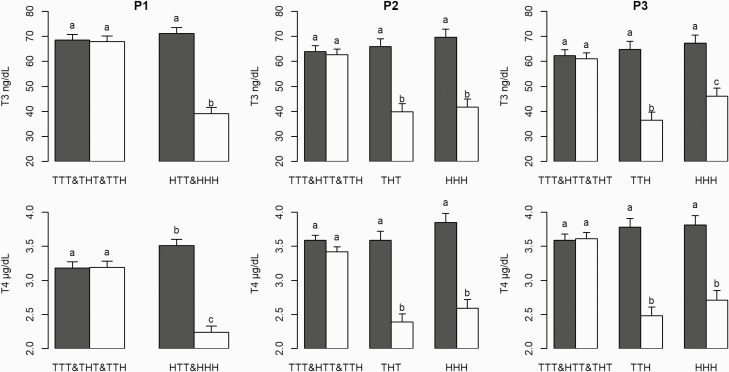
Effects of the temperature treatment and day (day −1: black bars, day 2: white bars) on plasma thyroid hormone concentrations in the first (P1), second (P2), and third (P3) experimental periods. Error bars indicate the standard error of least square mean. ^a,b^: bars with different superscripts differ significantly between days −1 and 2 (*P* < 0.05).

Regardless of the period, RR was significantly higher on days 0, 1, and 4 in HS groups (Fig. 4). The mean RR measured at 32 °C in HS groups was twice as high as that in groups kept in thermoneutral conditions from day 0 to 4 (86 vs. 37, 80 vs. 39, and 71 vs. 36 breaths/min in P1, P2, and P3, respectively). The effect of period on thermoregulatory responses during HS was tested using data from the HHH group (Fig. 4). Compared to the reference value (i.e., the mean RR of days −3 and −1), the increase in RR during the 5-d HS challenge was lower (*P <* 0.05) in P3 than in P1 and P2, especially on days 0 and 4 (+27 and +25 breaths/min vs. +46 and +40 breaths/min, respectively). During HS challenges, HS pigs had higher ST than pigs kept in thermoneutral conditions in P1, P2, and P3 (Fig. 5). Regardless of the period, ST was highest after 1 d of exposure to 32 °C (day 1) and did not significantly change from day 1 to 4. Once the HS challenge ended, HS pigs had lower ST than pigs kept in thermoneutral conditions, especially in P2 and P3. In the HHH group, this lower ST extended from the end of P2 to the beginning of P3 (Fig. 5). Regardless of the duration of exposure to 32 °C, the mean ST of the HHH group was lower in P3 than in P1 (35.8 vs. 36.9 °C, respectively; *P <* 0.01), with an intermediate value in P2 (36.3 °C; Fig. 5). Dynamics of T_core_ during the experiment varied among groups (Fig. 6A). In the TTT group, T_core_ linearly decreased throughout the experiment. Regardless of the period, exposure to HS challenges resulted in significant increases in T_core_, followed by a gradual decrease after 1–2 d at 32 °C. In P1, T_core_ did not differ among groups kept at 22 °C (TTT, THT, and TTH; *P >* 0.05). HTT and HHH groups had similar changes in T_core_ (*P >* 0.05) and showed a biphasic response, with a linear increase of 0.48 °C/d followed by a decrease of 0.30 °C/d after 1.5 d of exposure to 32 °C (Table 3). T_core_ reached a minimum 6.3 d after the beginning of the HS challenge. The mean T_core_ from day 6 to 10 was significantly lower in the H group than in the T group (38.5 vs. 38.8, respectively; *P <* 0.05). For P2, *y*_0_ and td_1_ were significantly higher in the THT group than in the TTT group (Table 3). Unlike in the TTT group, the mean T_core_ calculated from day 6 to 10 in the THT group did not differ from those in the TTT, HTH, and TTH groups (38.6 vs. 38.5, respectively; *P >* 0.05). In P3, the HHH group had a significantly lower *y*_0_ and mean T_core_ from day 6 to 10 than the HTH group (38.7 vs. 39.1, respectively, for *y*_0_ and 38.1 vs. 38.4, respectively, for T_core_; both *P <* 0.05). For the effect of period on T_core_ dynamics of the HHH group, *y*_0_ decreased significantly from P1 to P3, but the other parameters (*v*_1_, td_1_, *v*_2_, and td_2_) remained unaffected (Table 3). Plasma thyroid hormone levels (T3 and T4) varied by group and period (Fig. 7). Regardless of the period, T4 and T3 levels significantly decreased (*P <* 0.001) in the HS groups. In P3, T3 and T4 levels after 2 d of exposure to 32 °C were higher in the HHH group than in the TTH group (46.1 vs. 35.5 ng/dL, respectively, *P <* 0.01 for T3, and 2.71 vs. 2.48 µg/dL, respectively, *P =* 0.14 for T4).

## DISCUSSION

In temperate countries, summer heat waves are projected to become more frequent and severe due to climate change. Heat waves are defined as a number of consecutive days (at least 3–5) in which the ambient temperature exceeds the upper limit of the thermoneutral zone during both day and night. Heat waves are associated with reduced animal productivity and welfare, which results in animal mortality and lower performance (Lees et al., 2019). Surprisingly, few published studies have considered the effects of acute HS on pig performance and thermoregulatory responses (Abuajamieh et al., 2018; Mayorga et al., 2018). Most studies on the impact of HS have focused on chronic exposure (Renaudeau et al., 2011).

T_core_ varies as a function of the heat accumulated and dissipated between the animal and its environment. Therefore, these changes are a reliable indicator of heat storage and disrupted homeostasis. As previously described for rodents and pigs, thermoregulatory responses during the 5-d HS challenge was biphasic, with a short-term heat acclimation (STHA) phase characterized by a rapid thermoregulatory response, followed by a medium-term heat acclimation (MTHA) phase characterized mainly by a gradual decrease in T_core_ (Horowitz, 2002; Renaudeau et al., 2010). Based on measuring this response in the present study, the threshold day that indicated the beginning of the MTHA varied from 1.3 to 2.0 d, which agrees with previous studies (Renaudeau et al., 2010). During STHA, the sharp increase in RR was the main pathway of heat loss, and reducing ADFI was the main adaptation response to decrease metabolic heat production. However, these mechanisms were not sufficient to offset the heat load, which explains the rapid increase in T_core_ during STHA. The gradual decrease in T_core_ after the threshold day indicates that pigs were able to prevent an increase in body temperature. During MTHA, the decrease in RR indicates that pigs did not acclimate to the HS challenge by increasing evaporative heat losses. In fact, this acclimation response in pigs seems to be explained mainly by a decrease in resting heat production (Giles, 1992; Renaudeau et al., 2013). In the study of Renaudeau et al. (2013), we assumed that this greater heat tolerance enabled pigs to increase their feed intake gradually.

As expected, the HS challenge significantly decreased ADFI and ADG compared to those in thermoneutral conditions, and these responses varied by age (see below). The negative effect of high ambient temperature on ADFI is extensively described in the literature and is considered the main adaptive response for reducing heat production (Renaudeau et al., 2011; Baumgard and Rhoads, 2013). This reduced ADFI in HS animals decreased the amount of nutrients available for lean and fat deposition and decreased the growth performance. Expressed as a percentage of the performance observed during the prechallenge period, the HS challenge generally had more impact on ADG than on ADFI. In connection with the increase in F:G during the HS challenge, the greater effects of HS on ADG could be related to the “dilution” of the amount of energy available for growth due to the energy required for maintenance. However, the HS challenge likely had less effect on lean and fat deposition since some of the BW gain during the HS challenge was due to a difference in gut fill during weighing.

Compensatory growth after a period of undernutrition is a consistent feature of domestic animals. In pigs, the compensatory growth during a refeeding period depends on the severity and time of restriction (Lister and McCance, 1967; Lovatto et al., 2006), but it is generally accompanied by a distinct increase in voluntary feed intake. In the present study, the lack of compensatory growth was related to the pigs’ inability to increase their feed intake during the 7-d recovery period. Regardless of the period, HS pigs required 2–5 d to recover a feeding level similar to that of the non-HS pigs. These results generally agree with those of Rauw et al. (2017) and Mayorga et al. (2018). The latter study also examined compensatory responses during recovery in a pair-fed group of pigs previously housed in thermoneutral conditions. Unlike those of the HS pigs, the ADFI and ADG of pair-fed pigs significantly increased during recovery, suggesting that HS has specific effects on the mechanisms that underlie compensatory growth (Mayorga et al., 2018). Overall, the absence of complete recovery in feed intake and growth following an acute HS challenge indicates that physiological disturbances that occur during it have long-lasting effects on the growth of pigs. As mentioned by previous studies in swine, acute hyperthermia has transient negative consequences on intestinal morphology, integrity, and permeability (Baumgard and Rhoads, 2013; Liu et al., 2016). Thus, it can be hypothesized that these digestive disorders would limit the appetite during the recovery period. The inability of the HHH pigs to recover completely after three HS challenges may explain why they had lower ADG and final BW than the other groups.

In the present study, the effects of age and live BW on pig responses to the HS challenge were evaluated by comparing performances and physiological traits of the HTT, THT, and TTH groups. Compared to the 3-d period before the HS challenge, the decrease in ADFI during the 5-d period at 32 °C gradually increased as BW increased (−12%, −22%, and −26% in P1, P2, and P3, respectively). This result differs from those of Rauw et al. (2017), who observed that ADFI decreased the most during the first of three HS challenges. In the present study, the longer td_1_ for ADFI in P3 and P2 than in P1 seemed to confirm that older pigs were more susceptible to HS. In agreement with the effect of age on the decrease in ADFI in response to HS, much larger decreases in ADG were observed in P2 and P3 (−43% and −72%, respectively) than in P1 (−12%). These greater effects on growth performance could be due to the increase in maintenance energy requirements as BW increases and the subsequent decrease in the amount of energy available for growth. For T_core_, td_1_ and the increase in body temperature from *y*_0_ and td_1_ did not differ among HS challenges. Similar results were observed for the RR. This confirms that reducing ADFI to decrease metabolic heat production is the main pathway that pigs use to control body temperature during HS.

One hypothesis in the present study was that single or repeated HS challenges would help the animals respond to another HS challenge. Few studies address this topic for pigs. In the present study, pigs in the HHH group had a slightly lower decrease in ADFI and a lower increase in T_core_ during the second and third HS challenges than “unacclimated” pigs in the THT and the TTH groups, respectively. Similarly, repeated exposure to hyperthermia in humans (Périard et al., 2015) and rodents (Sareh et al., 2011) results in heat acclimation that is characterized by a lower increase in T_core_ in acclimated individuals when the HS challenge begins. Sareh et al. (2011) suggest that this apparent reduced susceptibility to HS is related mainly to improved heat elimination, which is reflected by an increase in sweating. According to Horowitz (2016), increased tolerance to a new HS challenge would decrease the T_core_ threshold for the onset of the acclimation response. The lower decrease in td_1_ during P2 in the HHH group compared to that in the THT group seems to confirm this hypothesis; however, the comparison of the HHH and HHT groups does not support it. In addition, the lower increase in T_core_ during STHA in HHH pigs could also be due to their relative hypothermia during the prechallenge period. Thus, it can be hypothesized that previous heat-induced hypothermic responses would help animals respond to new HS challenges. Postchallenge hypothermia was observed, especially in P1 for HTT pigs and in P1, P2, and P3 for HHH pigs. This heat-induced hypothermia agrees with results observed for poultry (Deaton et al., 1976; Teeter and Belay, 1996) and rodents (Wilkinson et al., 1988; Leon et al., 2005). Leon et al. (2005) suggested that heat-induced hypothermic responses could be interpreted either as an unregulated event due to direct thermal damage to homoeostatic sites or result of adaptive responses during the previous HS challenge. As shown by Liu et al. (2011), the depth and duration of heat-induced hypothermia during recovery is directly related to the severity of HS. The hypothermia after acute hyperthermia was recently also reported in pigs orally administered a temperature sensor for a continuous measurement of T_core_ (Kpodo et al., 2020). In contrast, Abuajamieh et al. (2018) and Mayorga et al. (2018) did not observe hypothermia in pigs during the recovery period, but they measured rectal temperature only during the daytime (0600, 1200, and 1800 h) after 3–7 d of exposure to elevated temperatures. In the present study, twice-daily rectal temperature measurements also failed to show heat-induced hypothermia (0900 and 1600 h; results not shown). In fact, according to the diurnal variation in T_core_ during the postchallenge period, hypothermia was due mainly to a large difference in nocturnal T_core_ (especially from 2100 to 0700 h) between HS and non-HS pigs. These observations emphasize the importance of continual measurements of T_core_ to assess the physiological status of animals accurately. Interestingly, our results indicated that heat-induced hypothermic responses depended on age: hypothermia during the recovery period was observed in P1 for the HS groups, but it was observed in P2 and P3 only when pigs were previously exposed to an HS challenge during P1. To date, no clear explanation exists for this result, and further studies are needed to understand the underlying mechanisms better.

## CONCLUSION

Studies of impacts of acute heat challenges that mimic summer heat waves remain rare for pigs. Exposure to a 5-d HS challenge caused a large decrease in ADFI and ADG and an increase in T_core_. These responses differed by the age of the animal. Our results suggest that summer heat waves can have long-lasting effects on performances and physiological responses, which thus require new adaptation strategies. Preliminary exposure to an HS challenge may help animals become less sensitive to new climate disturbances.
